# Improving the thermostability of *Pseudoalteromonas Porphyrae* κ-carrageenase by rational design and MD simulation

**DOI:** 10.1186/s13568-024-01661-z

**Published:** 2024-01-20

**Authors:** Yuyan Sang, Xiaoyi Huang, Hebin Li, Tao Hong, Mingjing Zheng, Zhipeng Li, Zedong Jiang, Hui Ni, Qingbiao Li, Yanbing Zhu

**Affiliations:** 1 College of Ocean Food and Biological Engineering, Jimei University, 361021 Xiamen, China; 2 Department of Pharmacy, Xiamen Medical College, 361008 Xiamen, China; 3 Fujian Provincial Key Laboratory of Food Microbiology and Enzyme Engineering, 361021 Xiamen, China; 4 Research Center of Food Biotechnology of Xiamen City, 361021 Xiamen, China

**Keywords:** Mutant κ-carrageenase, Folding free energy change, Molecular dynamic simulation, Thermostability improvement

## Abstract

The industrial applications of the κ-carrageenases have been restricted by their poor thermostability. In this study, based on the folding free energy change (ΔΔG) and the flexibility analysis using molecular dynamics (MD) simulation for the alkaline κ-carrageenase KCgCD from *Pseudoalteromonas porphyrae* (WT), the mutant S190R was identified with improved thermostability. After incubation at 50 °C for 30 min, the residual activity of S190R was 63.7%, 25.7% higher than that of WT. The *T*_m_ values determined by differential scanning calorimetry were 66.2 °C and 64.4 °C for S190R and WT, respectively. The optimal temperature of S190R was 10 °C higher than that of WT. The κ-carrageenan hydrolysates produced by S190R showed higher xanthine oxidase inhibitory activity compared with the untreated κ-carrageenan. MD simulation analysis of S190R showed that the residues (V186–M194 and P196–G197) in F5 and the key residue R150 in F3 displayed the decreased flexibility, and residues of T169–N173 near the catalytic center displayed the increased flexibility. These changed flexibilities might be the reasons for the improved thermostability of mutant S190R. This study provides a useful rational design strategy of combination of ΔΔG calculation and MD simulation to improve the κ-carrageenase’s thermostability for its better industrial applications.

## Key points

*Mutant κ-carrageenase S190R is identified by rational design based on ΔΔG and MD simulation.*

*Mutant κ-carrageenase S190R increases the thermostability.*

*Mutant enzyme-treated κ-carrageenan exhibits high xanthine oxidase inhibitory activity.*

## Introduction

Carrageenan is a linear sulfated polysaccharide extracted from certain red seaweeds of *Rhodophyceae* class, and it is composed of D-galactose alternately linked by α-1,3 and β-1,4 linkages (Zhu et al. 2018). According to their different degrees and positions of sulfation substitution, carrageenan can be classified into different subtypes. Among them, the κ-carrageenan is composed of alternating units of β-D-galactose-4-sulfate and 3,6-anhydro-α-D-galactopyranose by α-1,3 and β-1,4 glycosidic bonds connections (Zia et al. 2017). It is widely used as a stabilizer, thickener, or gelling agent in food industry. Additionally, various biological activities including antioxidant, immunostimulatory, and anticoagulant of κ-carrageenan have been reported (Jiang et al. 2021). However, the κ-carrageenan has the high molecular weight, high viscosity, and low solubility, resulting in the its poor bioavailability. The applications of κ-carrageenan are greatly limited in the pharmaceutical industry. Compared to the κ-carrageenan, the κ‑carrageenan oligosaccharides have lower viscosity, excellent solubility, and good tissue permeability. It has been reported that the bioavailability and biological activities can be enhanced by the degradation of the κ-carrageenan (Guo et al. 2022), making the oligosaccharides great application potentials in the medicine (Cicinskas et al. 2020), cosmetics (Pangestuti et al. 2021), and agriculture (Zhu et al. 2021a). The bioactivities of κ-carrageenan oligosaccharides include the anti-tumor, anticoagulant, anti-inflammatory, antithrombotic, and viral inactivation activities (Guo et al. 2022). The κ-carrageenan oligosaccharides with different structure, polymerization degree, and sulfate content can be obtained by different preparation methods, including chemical, physical, and enzymatic approaches (Bouanati et al. 2020; Guo et al. 2022; Sun et al. 2010). Among them, the enzymatic hydrolysis has been a promising and preferred method for carrageenan oligosaccharides production because of its strong specificity and mild reaction conditions (Cheong et al. 2018).

The κ-carrageenase (EC 3.2.1.83), a member of glycoside hydrolase family 16 (GH16), is capable of breaking the β-1,4 linkages in κ-carrageenan to produce even-numbered κ-carrageenan oligosaccharides (Zhu et al. 2018). In addition to producing the biologically active κ-carrageenan oligosaccharides, the κ-carrageenase can also be applied as a component of detergents in the textile industry, as a saccharifying agent in bioethanol production, as well as to isolate the algae protoplast (Ghanbarzadeh et al. 2018). High concentrations of κ-carrageenan have high viscosity at room temperature, that can reduce the catalytic efficiency of the κ-carrageenase. High temperature operation will reduce the viscosity of κ-carrageenan and reduce the risk of pollution, thus promoting the increase of substrate solubility, diffusion speed, and mass transfer (Li et al. 2019). In addition, high temperature leads to the conformational transformation of polysaccharides, followed by helical dissociation. This makes it easier for the carrageenase to contact with the substrate, thereby acting on the polysaccharides (Ma et al. 2013). Therefore, the κ-carrageenase with good thermostability is very beneficial for the κ-carrageenan depolymerization process (Li et al. 2019). However, the optimal temperature for most carrageenases is around 30–40 °C (Zhu et al. 2018). Many strategies have been developed for screening enzymes with good thermostability (Lee et al. 2017; Wu et al. 2023), and protein engineering is the commonly used and effective way (Nezhad et al. 2023). Among the approaches for protein engineering, rational design has been a popular method to obtain enzymes with improved thermostability (Liu et al. 2019), mainly including homologous sequence alignment, protein surface charge engineering, disulfide bond design, proline and glycine design, protein unfolding free energy design, temperature factor design, and unstable region anchoring (Wu et al. 2023). Molecular dynamics (MD) simulation provides a powerful technique for understanding the relationships between protein sequences, structures, dynamics, interactions, and functions (Chen et al. 2021). The bioinformatics software FoldX can be used to predict the impact of protein mutations on folding free energy change (ΔΔG) based on protein sequences or three-dimensional structures (Wu et al. 2023). Analyzing the structure of enzymes and identifying specific regions or residues of enzymes can greatly reduce the numbers of the predicted mutations. By combining MD simulation and FoldX, protein residues with low ΔΔG values and high RMSF values can be screened for mutation, and the enzymatic properties of the mutants can be further determined.

The catalytic domain of *Pseudoalteromonas porphyrae* LL1κ-carrageenase (GenBank accession number GU386342) has good enzymatic activity against κ-carrageenan at 40 °C and pH 8.0, and show an excellent stability over a wide alkaline pH range from 7.0 to 10.0 (Zhao et al. 2018). The enzyme exhibits stability below 35 °C, but its thermostability decreases rapidly when the temperature is above 45 °C (Zhao et al. 2018). In this study, the catalytic domain of *P. porphyrae* κ-carrageenase (named KCgCD) was expressed in *E. coli* BL21 (DE3). Based on the structure of KCgCD, FoldX 5.0 and MD simulation were combined to predict the hot spot mutations with improved thermostability by calculating the ΔΔG and root mean square fluctuation (RMSF) of the enzyme residues. Heat-resistant variants of KCgCD were screened from the predicted candidates, and then the possible mechanism for the improved thermostability was elucidated through bioinformatics analysis. At last, the mutant κ-carrageenase was applied for the enzymatic degradation of κ-carrageenan and the xanthine oxidase inhibitory activities of the degradation products were evaluated.

## Materials and methods

### Structure and molecular docking analysis

Gene and protein sequences of κ-carrageenase were analyzed by the DNAMAN 5.1 program (Lynnon BioSoft, Quebec, Canada). The 3D structure of the catalytic domain of κ-carrageenase from *Pseudoalteromonas porphyrae* LL1 was constructed by the online AlphaFold2 system. Intramolecular interactions were analyzed using Ring-3.0 online server (https://ring.biocomputingup.it/) (Clementel et al. 2022). AutoDock Vina 1.1.2 software was used for the molecular docking of κ-neocarratetraose and κ-carrageenase (Trott and Olson 2010). The hydrogen bonds and hydrophobic interactions between κ-neocarratetraose and κ-carrageenase were analyzed using LigPlot^+^ online server (Laskowski and Swindells 2011). Protein structure was visualized and analyzed by Discovery Studio 2019 (BIOVIA, San Diego, CA, USA) and PyMol Molecular Graphics System (DeLano Scientific, San Carlos, CA, USA).

### Molecular dynamics (MD) simulation

Under the force field of GROMOS96 54a7, GROMACS 5.1.4 software was used to conduct 20 ns unconstrained molecular dynamics simulation of κ-carrageenase. The protein was placed in a periodic cubic solvent box and buffered at a distance of 1 nm in each dimension. Charge balance was achieved by adding Na^+^ or Cl^−^ ions. Using LINC as constraint algorithm, V-rescale method was selected for temperature control. The pressure control was performed using the Parrinello-Rahman method. After the simulation was completed, the built-in tool of the software was used to analyze the trajectory. The stability of the simulated conformation was demonstrated using the values of root mean square deviation (RMSD), root mean square fluctuation (RMSF), and radius of gyration (R_g_).

### Computational prediction for κ-carrageenase thermostability

FoldX 5.0 was used to estimate the mutational impact as a change in folding free energy for κ-carrageenase KCgCD (Delgado et al. 2019). In the output results, a negative value of ΔΔG indicates a more stable mutation, while a positive value indicates an unstable mutation (Komor et al. 2012). Here we selected the mutants with the predicted ΔΔG values below − 0.5 kcal/mol. The enzyme was predicted for residue conservation using the ConSurf server (http://consurfdb.tau.ac.il/) (Ben Chorin et al. 2020; Celniker et al. 2013). The conservative residues and amino acid residues within the range of 5 Å of the predicted catalytic triad (E162-D164-E167) of KCgCD were excluded. In the meanwhile, the GROMACS 5.1.4 program (http://www.gromacs.org/) was applied for the calculation of RMSF values of KCgCD at 323 K. The amino acid residues with RMSF > 0.3 were selected.

### Construction of κ-carrageenase gene and site-directed mutagenesis

The wide-type gene of the catalytic domain of *P. porphyrae* κ-carrageenase (WT) was synthesized by BGI Genomics Co., Ltd. (Shenzhen, China) and inserted into the pET-28a (+) vector (Novagen, Madison, WI, USA). The recombinant plasmid was introduced into *E. coli* BL21 (DE3) for enzyme expression. The recombinant plasmid containing the WT gene was used as the template, and the primers were designed according to the requirements of the kit (Table 1). Then the Mutant Express II rapid mutation kit V2 (Vazyme, Nanjing, China) was used for site-directed mutagenesis. PCR reaction parameters: 95 °C for 30 s; 95 °C for 15 s, 62 °C for 15 s, 72 °C for 1 min, 30 cycles; 72 °C for 5 min, stored at 4 °C. After the reaction, 2 µL *Dpn* I (10 U/µL) was added and the mixture was digested at 37 °C for 1 h. The cyclization products were transformed into *E. coli* BL21 (DE3), and the recombinant plasmid containing the mutated κ-carrageenase gene was extracted for sequencing.

**Table 1:** Primers for site-directed mutagenesis

Primers	Primer sequences (5'-3')	T_m_ (°C)
S76M-F	5’- CCACCGTAatgAATGGTAAGTTGAAGTTAACAACTAAGCG − 3’	63.1
S76M-R	5’- ACCATTcatTACGGTGGCATTTTCATTACGC − 3’	62.7
G96M-F	5’- GGGATatgTGTAATCAGCAGCAGGTAGCAAA − 3’	60.6
G96M-R	5’- CTGATTACAcatATCCCAAAATGTACGATTATGTGTTT − 3’	60.7
C97K-F	5’- GGATGGCaaaAATCAGCAGCAGGTAGCAAATTATC − 3’	61.6
C97K-R	5’- GCTGATTtttGCCATCCCAAAATGTACGATTAT − 3’	60.2
Q100M-F	5’- AATCAGatgCAGGTAGCAAATTATCCTCTGTACTATACA − 3’	61.3
Q100M-R	5’- GCTACCTGcatCTGATTACAGCCATCCCAAAATG − 3’	61.4
T151S-F	5’- AGATCGCagcTTAACTGAAAATGGTGATGTACAGTACAG − 3’	61.0
T151S-R	5’- CAGTTAAgctGCGATCTATTGTGCTATACATCCAA − 3’	60.8
L152R-F	5’- TCGCACAcgtACTGAAAATGGTGATGTACAGTACAGTG − 3’	61.2
L152R-R	5’- TTTCAGTacgTGTGCGATCTATTGTGCTATACATCC − 3’	62.7
T153L-R	5’- GATCGCACATTActgGAAAATGGTGATGTACAGTACAGTGAAA-3’	61.3
T153L-F	5’- TCcagTAATGTGCGATCTATTGTGCTATACAT-3’	60.1
E154R-F	5’- AACTcgtAATGGTGATGTACAGTACAGTGAAATTG − 3’	61.2
E154R-R	5’- CATCACCATTacgAGTTAATGTGCGATCTATTGTGCTATAC − 3’	60.5
N155L-F	5’- AACTGAActgGGTGATGTACAGTACAGTGAAATTGATG − 3’	61.4
N155L-R	5’- CATCACCcagTTCAGTTAATGTGCGATCTATTGTGC − 3’	62.9
S190R-F	5’- AAATGGCcgtCCTACTTGGATGCGACCGG − 3’	61.1
S190R-R	5’- AAGTAGGacgGCCATTTTTAACGACAATGTTATGT − 3’	60.0
T192R-F	5’- AATGGCTCTCCTcgtTGGATGCGACCGGGTAGTG − 3’	62.2
T192R-R	5’- CAacgAGGAGAGCCATTTTTAACGACAA − 3’	60.3
G197M-F	5’- TTGGATGCGACCGatgAGTGCGCCAGAAACAAATCATA − 3’	60.2
G197M-R	5’- TcatCGGTCGCATCCAAGTAGGAG − 3’	60.5
E201P-F	5’- CGCCAccgACAAATCATAATGGCTATCACTTACCG − 3’	62.6
E201P-R	5’- ATGATTTGTcggTGGCGCACTACCCGGTCG − 3’	65.7
T202M-F	5’- GCGCCAGAAatgAATCATAATGGCTATCACTTACCGTT − 3’	60.3
T202M-R	5’- TGATTcatTTCTGGCGCACTACCCGG − 3’	62.5
E279D-F	5’- ATCAGCAgatGGCTTTCCTACCTCAATGGAAGT − 3’	60.9
E279D-R	5’- GAAAGCCatcTGCTGATTTATTAGCAGATGGGTAA − 3’	60.6

*Notes:* Lowercase letters represented the mutation sites. The reverse primers were reversely complementary to the exact sequence of the forward primers.

### Protein expression and purification

The engineered *E. coli* BL21 cells containing the κ-carrageenase gene were cultured at 37 °C in 300 mL LB medium containing 50 µg/mL kanamycin. After the OD_600_ reached 0.8, these cells were induced with 0.075 mM isopropyl-β-D-thiogalactopyranoside (IPTG) at 4 °C for 16 h. After centrifugation (5000× *g*, 5 min), the cell lysis was performed by sonication. The lysed products were subjected to centrifugation (12,000× *g*, 20 min) at 4 °C, and the crude κ-carrageenase extracts were obtained by collecting the supernatant. The crude protein was purified using Ni Sepharose 6 Fast Flow (GE Healthcare Life Science, Uppsala, Sweden) affinity chromatography according to the method described before (Li et al. 2022). The purified enzyme was dialyzed against 50 mM sodium phosphate buffer (pH 8.0). The purity of recombinant κ-carrageenase was evaluated by SDS-PAGE. The purified enzyme was stored at − 20 °C for further analysis.

### Activity assay of κ-carrageenase

The activity of κ-carrageenase was determined by measuring the concentration of released reducing sugars using 3,5-dinitrosalicylic acid (DNS) method (Miller 1959). The κ-carrageenan (TEXOMES, Catalonia, Spain) was dissolved in 50 mM sodium phosphate buffer (pH 8.0) to prepare 0.5% κ-carrageenan substrate solution. After 10 µL of the κ-carrageenase (30 ng/µL) was added to 490 µL of the substrate solution, the reaction was performed at the optimal temperature for 15 min. The DNS solution (500 µL) was added and the mixture was boiled to end the reaction. After cooling to room temperature, the amount of reducing sugar was determined at 520 nm by a Cary 50 spectrophotometer (Varian, Palo Alto, CA, USA). The measurement was obtained from three biological replicates. One unit of the κ-carrageenase was defined as the amount of enzyme that produced 1 µmol of galactose per minute under the assay conditions.

### Effects of temperature and pH on the enzymatic activity and stability

The activity of κ-carrageenase was measured at different temperatures (30–60 °C) to determine its optimal reaction temperature. To determine the enzymatic thermostability, the κ-carrageenase was preincubated at different temperatures (45, 50, 55, and 60 °C) for 30 min. The residual activity was measured at the optimal temperature and pH 8.0. The relative activity of enzyme without thermal treatment was defined to be 100%. In order to determine the optimal reaction pH for κ-carrageenase, the enzyme activity was measured in 50 mM of different buffers with the pH range of 4.0–10.0. The buffers of citrate-Na_2_HPO_4_ (pH 4.0–6.0), sodium phosphate (pH 6.0–8.0), Tris-HCl (pH 8.0–9.0), and glycine-NaOH (pH 9.0–10.0) were included in the assays.

### Differential scanning calorimetry analysis of κ-carrageenase

Melting temperature (*T*_m_) of κ-carrageenase was measured using Discovery SDT 650 (Taber, New York, USA). Pure metal indium (99.99%) was used for the calibration of temperature and enthalpy values of the instrument. The κ-carrageenase (3.0 ± 0.1 mg) was heated from 20 to 100 °C at a rate of 1 °C/min. The experiment was carried out under dry N_2_ with a purge gas of 20 mL/min and a protective gas of 60 mL/min.

### Kinetic parameters determination of κ-carrageenase

The κ-carrageenase was reacted with the κ-carrageenan substrate with different concentrations to determine the enzymatic activity at the optimal temperature and pH 8.0 for 15 min, respectively. The values of Michaelis-Menten substrate affinity constant (*K*_m_) and the maximum velocity (*V*_max_) were obtained based on the double reciprocal curves of enzyme activities at different concentrations of the substrate.

### Fluorescence spectroscopy analysis of κ-carrageenase

The fluorescence spectrum of κ-carrageenase (0.2 mg/mL) was acquired by a Cary Eclipse fluorescence spectrophotometer (Agilent Technologies, Palo Alto, CA, USA) at room temperature. The experiment was conducted under the excitation wavelength of 317 nm, with a scanning range of 330–500 nm. The excitation slit width and emission slit width were both 5 nm. The average of the three measurements was taken as the experimental results.

### Preparation and determination of the enzymatic degradation products

To investigate the effect of mutation on the enzymatic hydrolysis products, 3 U of the purified WT or its mutant was reacted with 15 mL of 0.5% κ-carrageenan substrate solution at 40 °C. The reducing sugar content was measured at regular intervals. After 2 h of incubation, the reducing sugar content became constant. Then the mixture was heated to boil and kept for 10 min. After cooling to room temperature, 3-fold volume of anhydrous alcohol were added to the system and the mixture was left overnight at 4 °C. After centrifugation (5000× *g*, 15 min) at 4 °C, the supernatant was collected, evaporated, and then freeze-dried to obtain the enzymatic hydrolysis products. In the negative ion mode, the digestion products were analyzed using the mass spectrometer (MS) analysis system TSQ Altis Triple Quadruple (Thermo Fisher Science, Waltham, MA, USA).

### Inhibitory effect of the enzymatic degradation products on xanthine oxidase (XOD) activity

The inhibitory effect of κ-carrageenan oligosaccharides on XOD activity was determined according to the pervious method with slight modifications (Qi et al. 2022). Firstly, 50 µL of κ-carrageenan oligosaccharides solution at different concentrations was mixed with 50 µL of 0.05 U/mL XOD (Macklin, shanghai, China) and incubated at 37 °C for 30 min. Then, 150 µL of xanthine at the concentration of 0.42 mM was added. At last, the absorbance was recorded at 292 nm every 40 s for 6 min. The inhibitory effect of κ-carrageenan oligosaccharides on XOD activity was calculated as (1–Rs/Rc)×100%, where Rs and Rc were the slopes of the reaction kinetic curves obtained from the reactions with and without inhibitors, respectively. The undigested κ-carrageenan was used as the control.

## Results

### Screening of mutants with improved thermostability

**Fig. 1:**
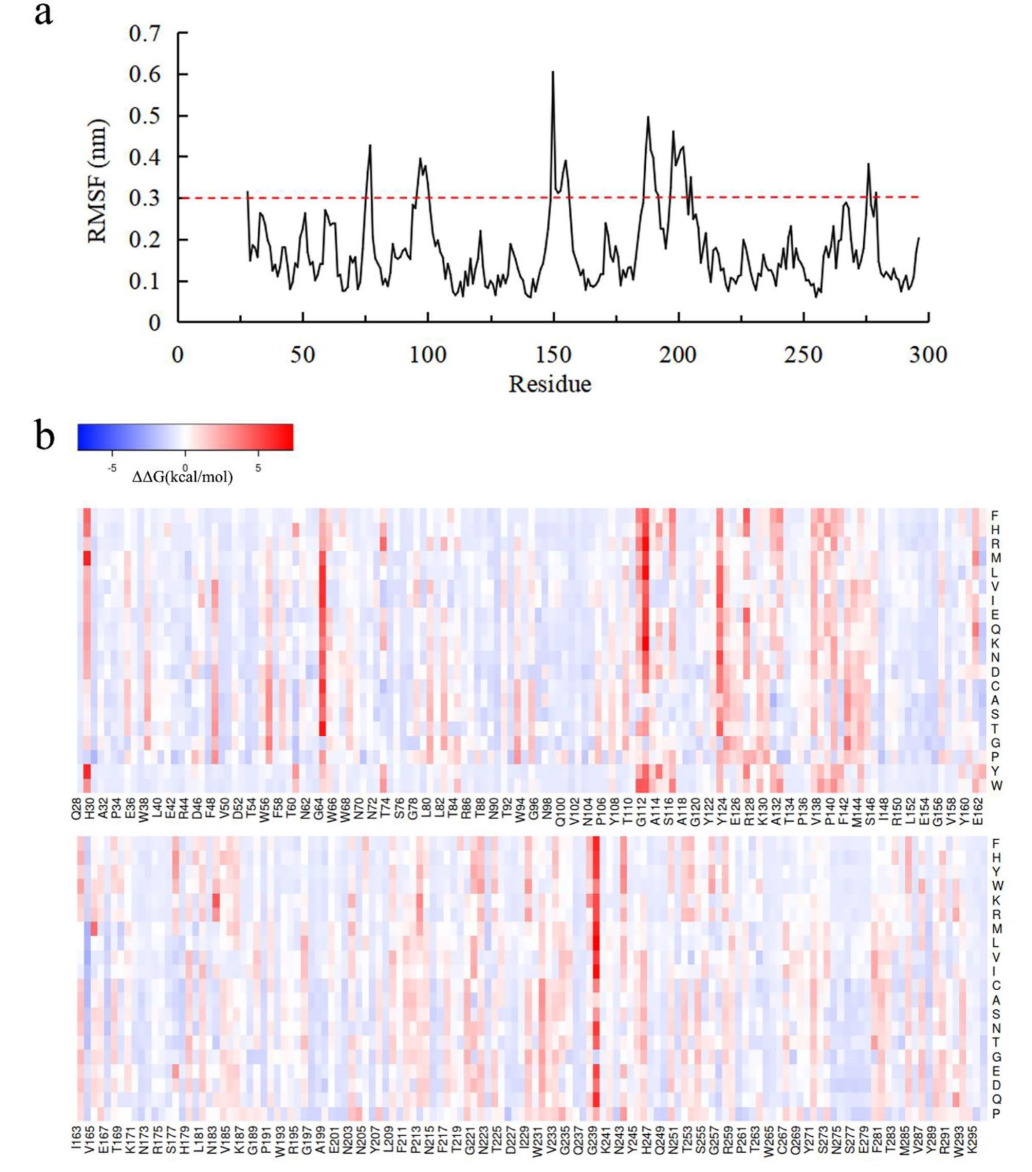
RMSF and ΔΔG values of wild-type κ-carrageenase. **(a)** RMSF values of WT at 323 K. **(b)** Heat map of WT regarding ΔΔG values. The bottom line displayed the sequence of WT, while the vertical axis displayed the other 20 possible amino acids at each position. The color bar represented the magnitude of the predicted change in thermostability. Stable mutations were indicated in blue, while unstable mutations were indicated in red. The darker the color, the higher the degree of the possible change in thermostability

**Fig. 2:**
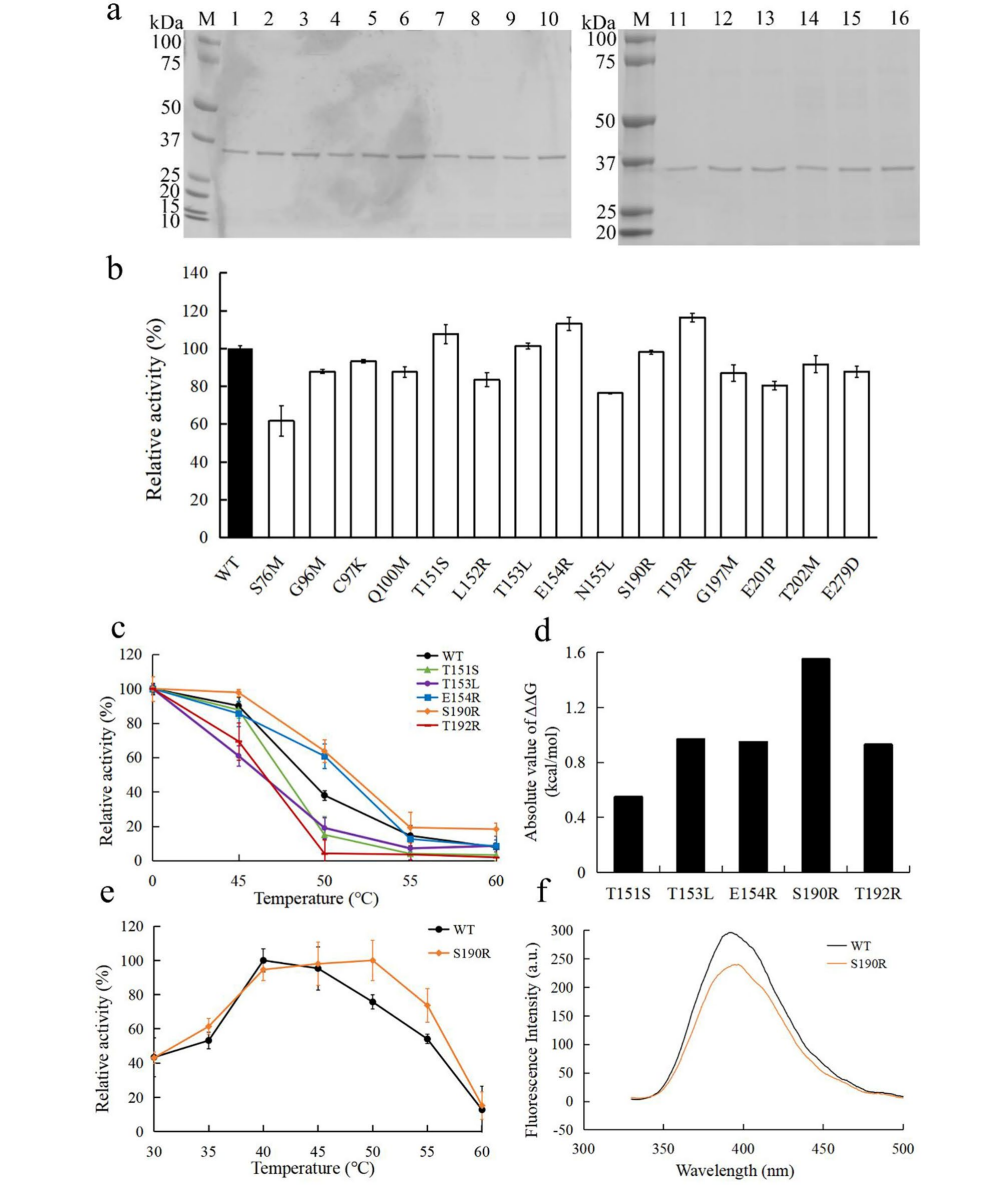
The enzymatic properties and fluorescence spectrum analysis of κ-carrageenases. **(a)** SDS-PAGE analysis of wild-type κ-carrageenanase and its mutants. Band M represented the standard protein marker, and bands 1–16 represented WT, S76M, G96M, C97K, Q100M, T151S, L152R, T153L, E154R, N155L, S190R, T192R, G197M, E201P, T202M, E279D, respectively. **(b)** The relative activities of 15 mutant κ-carrageenases. The enzyme activity of WT was defined as 100%. **(c)** Thermostability of 5 mutant κ-carrageenases (T151S, T153L, E154R, S190R, and T192R) after treatment at 45–60 °C for 30 min. **(d)** Absolute values of ΔΔG for 5 mutants of T151S, T153L, E154R, S190R, and T192R. **(e) **The optimal temperature of S190R and WT. **(f)** Fluorescence spectral analysis of S190R and WT.

The overall structure of *P. porphyrae* κ-carrageenase KCgCD was predicted to fold a β-sandwich formed by tightly packed and curved antiparallel β-sheets, with catalytic active centers (E162-D164-E167) located inside the pocket. As an important biophysical property of proteins, the values of ΔΔG reflect the overall stability of 3D structure of macromolecules (Buß et al. 2018b). Based on the 3D structure, conservation, flexibility (Fig. 1a), and ΔΔG energy calculation (Fig. 1b) analysis of WT, 15 mutants were selected for further screening, including S76M, G96M, C97K, Q100M, T151S, L152R, T153L, E154R, N155L, S190R, T192R, G197M, E201P, T202M, and E279D. After in vitro expression and purification, the mutants were analyzed using SDS-PAGE and showed a single band (about 35.0 kDa), which was consistent with that of WT (Fig. 2a). Compared with WT, the enzyme activities of T151S, E154R, and T192R were increased, the activities of T153L and S190R remained basically unchanged, while the activities of other mutants were decreased (Fig. 2b). Therefore, T151S, E154R, T192R, T153L, and S190R were selected for the subsequent thermostability measurements. Among them, the thermostability of S190R were significantly improved (Fig. 2c). After incubation at 50 and 60 °C for 30 min, the residual activities of S190R were 63.7% and 18.3%, respectively. They were 25.7% and 10.5% higher than that of WT, respectively. The temperature at which κ-carrageenase lost 50% enzyme activity after 30 min of heat treatment (*T*_50_^30^) was calculated. The *T*_50_^30^ value of S190R was 52.0 °C with 2.3 °C higher than that of WT (Table 2). The melting temperature (*T*_m_) refers to the temperature at which protein structure unfolds and loses its unique 3D structure (Elias et al. 2014). The differential scanning calorimetry (DSC) not only can be used to determine the equilibrium thermodynamic stability and folding mechanism of proteins, but also can be used to determine the proteins’ thermostability in a more qualitative manner (Johnson 2013). The results of DSC analysis displayed that the *T*_m_ values of S190R and WT were 66.2 °C and 64.4 °C, respectively (Table 2). These results demonstrated that the thermostability of mutant S190R was better than that of WT. The higher absolute values of ΔΔG in mutants, the greater possibility of thermostability improving through mutagenesis. In this study, the absolute ΔΔG value of S190R was the highest among the selected five mutants (Fig. 2d), approving the credibility of the mutant prediction by FoldX to a certain extent. At present, the modifications for the thermostability improvement of the κ-carrageenase rely solely on the server algorithm tools (Hong et al. 2023). In this study, the combined strategy of the server calculations and molecular dynamics simulation was used to screen and identify the mutants with improved thermostability of the κ-carrageenase KCgCD.

**Table 2:** Property comparisons of S190R and WT

	^a^Relative enzyme activity (%)	^b^T_opt_ (°C)	^c^pH_opt_	T_50_^30^ (°C)	T_m_ (°C)	K_m_ (mg/mL)	V_max_ (U/mg)
WT	100	40	8.0	49.7	64.4	2.87	625
S190R	98.1	50	8.0	52.0	66.2	7.00	1250

*Notes: *^a^Enzyme activity was measured using the standard measurement method described in “Materials and methods”, and the enzyme activity of WT was defined as 100%. ^b^T_opt_ standed for the optimal temperature. ^c^pH_opt_ standed for the optimal pH.

### Other enzymatic properties and fluorescence spectroscopy of S190R

The other enzymatic properties of the purified mutant S190R were also determined. The mutation of S190R had no effect on the optimal pH with the value of 8.0 (Table 2). However, the optimal reaction temperature for S190R was 50 °C, which was 10 °C higher than that of WT (Fig. 2e), indicating that the mutation of S190R helped improve the enzyme activity at the higher temperatures. As shown in Table 2, according to the Lineweaver-Burk plot method, the *K*_m_ values of WT and S190R were calculated to be 2.87 mg/mL and 7.00 mg/mL, while the *V*_max_ values of WT and S190R were calculated to be 625 U/mg and 1250 U/mg, respectively.

Fluorescence spectra can be used to obtain the structure information of biomolecules (Ghosh and Enderlein 2021; Samukaite-Bubniene et al. 2020). In the fluorescence spectroscopy analysis, as shown in Fig. 2f, the mutant S190R and WT shared the same maximum fluorescence emission wavelength at 390 nm, indicating that the tertiary structure of κ-carrageenase was not affected by the mutation from serine to arginine at the 190th site.

### LC-MS analysis of enzymatic degradation products

**Fig. 3:**
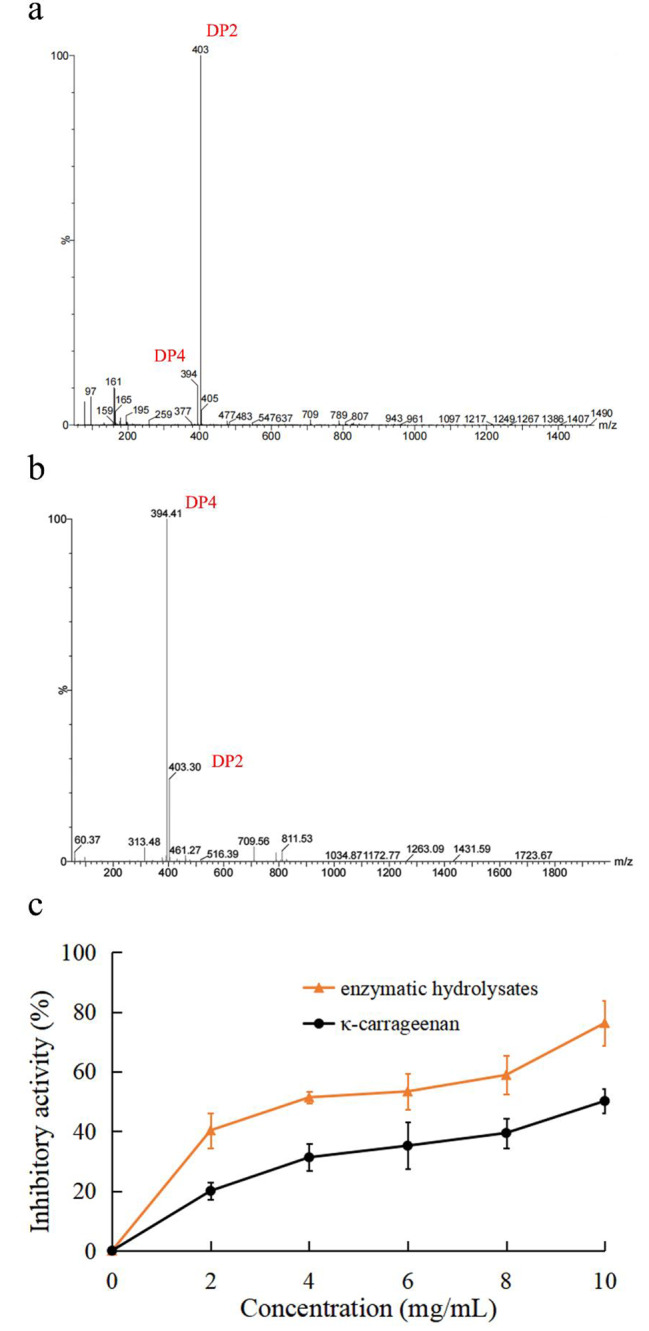
LC-MS analysis of the enzymatic hydrolysates and their inhibitory effects against xanthine oxidase activity. **(a)** LC-MS analysis of κ-carrageenan hydrolysates by S190R. **(b)** LC-MS analysis of κ-carrageenan hydrolysates by WT. **(c) **Xanthine oxidase inhibitory activity of κ-carrageenan enzymatic hydrolysates and untreated κ-carrageenan

The structures of the enzymatic degradation products can be analyzed by MS, with degree of polymerization (DP) deduced by mass-to-charge ratios (Hong et al. 2023). The mass spectrum peaks of mutant S190R included 394 *m/z* and 403 *m/z*, respectively, symbolizing [(An-G4S)_2_]^2−^ (κ-carrageenan tetrasaccharide) and [(An-G4S)]^−^ (κ-carrageenan disaccharide) (Sun et al. 2015). These results implied that enzymatic hydrolysis products of κ-carrageenan by S190R were disaccharides and tetrasaccharides (Fig. 3a), which was consistent with that of WT (Fig. 3b), indicating that the mutation at site 190 did not alter the degradation mode of the enzyme.

### Inhibitory effects of enzymatic degradation products against xanthine oxidase activity

The XOD inhibitory activity of κ-carrageenan and κ-carrageenan oligosaccharides obtained by S190R hydrolysis were evaluated. Compared with κ-carrageenan, the κ-carrageenan oligosaccharides showed stronger inhibitory effects against XOD activity (Fig. 3c). In the concentration range of 2.0–10.0 mg/mL, the inhibition rate of κ-carrageenan oligosaccharides to XOD activity was about 40.3–76.3%. The half inhibitory concentration (IC_50_) of enzymatic hydrolysis products was 3.7 mg/mL, much lower than 9.9 mg/mL of the untreated κ-carrageenan (Fig. 3c).

### Structure analysis of S190R

**Fig. 4:**
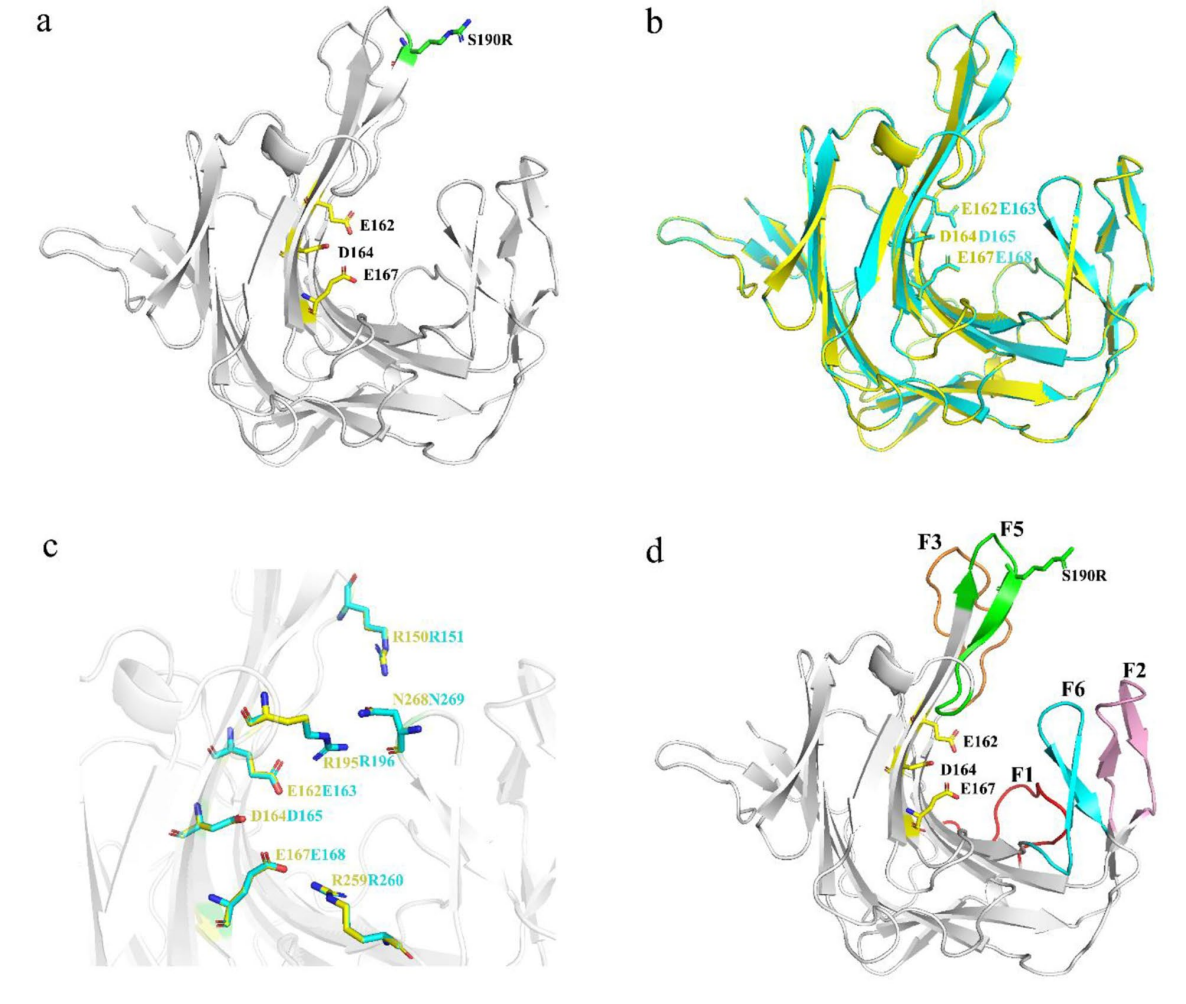
Structure analysis of the mutant κ-carrageenase S190R. **(a) **The three-dimensional structure of S190R. The catalytic triad was represented by yellow sticks and the mutation site was represented by a green stick.** (b)** Overlapping schematic diagram of the overall 3D models for S190R and Cgk-K142. The yellow model represented the S190R, the blue model represented Cgk-K142, and the catalytic triads of S190R and Cgk-K142 were represented by the yellow and blue sticks, respectively. **(c)** Overlapping schematic diagram of the important residues for S190R and Cgk-K142. The important residues of S190R and Cgk-K142 were represented by the yellow and blue sticks, respectively. **(d)** The model of the mutant κ-carrageenase S190R. The “finger” regions around the catalytic channel were numbered from F1 to F6. The catalytic triad E162-D164-E167 was marked in the form of yellow sticks

Compared with the structure of WT, the mutation at the 190th site did not cause significant structural changes. The structure of S190R still exhibited a β-sandwich structure (Fig. 4a). By protein sequence alignment, it was found that the structure of *P. porphyrae* κ-carrageenase mutant S190R in this study was very similar to that of κ-carrageenase Cgk-K142 from *Pseudoalteromonas tetraodonis* (GenBank accession number AB572925) (Kobayashi et al. 2012) with the sequence similarity of 90.51%. Their 3D structures overlapped well, including the predicted catalytic triad of the two κ-carrageenases (Fig. 4b) and other important residues (Fig. 4c). According to the structure of Cgk-K142 (Matard-Mann et al. 2017), five fingers could be predicted for S190R, including F1 (W56–W68), F2 (R91–Y105), F3 (T147–Q159), F5 (V186–G197), and F6 (A260–P272) (Fig. 4d). The mutation site 190 was located on the F5 finger, far away from the catalytic center (Fig. 4d). E162 and E167 were predicted to be the nucleophile and acid/base catalytic residues, respectively, and D164 was predicted to participate in proton trafficking, forming the catalytic triad of the enzyme. In addition, R195 (located in F5) and N268 (located in F6) in S190R were predicted to adopt the “closed” conformation with the existence of substrate, indicating that they might be involved in the formation of tunnel above the catalytic active site (Matard-Mann et al. 2017). Residue R150 (located in F3) was predicted to establish strong interactions with the substrate at the entrance of the catalytic groove (Matard-Mann et al. 2017). Residue R259 was predicted to be important in stabilizing the substrate in subsite − 1 through the interactions with the sulfate group (Matard-Mann et al. 2017).

### Molecular docking and interaction analysis of κ-carrageenase

**Fig. 5:**
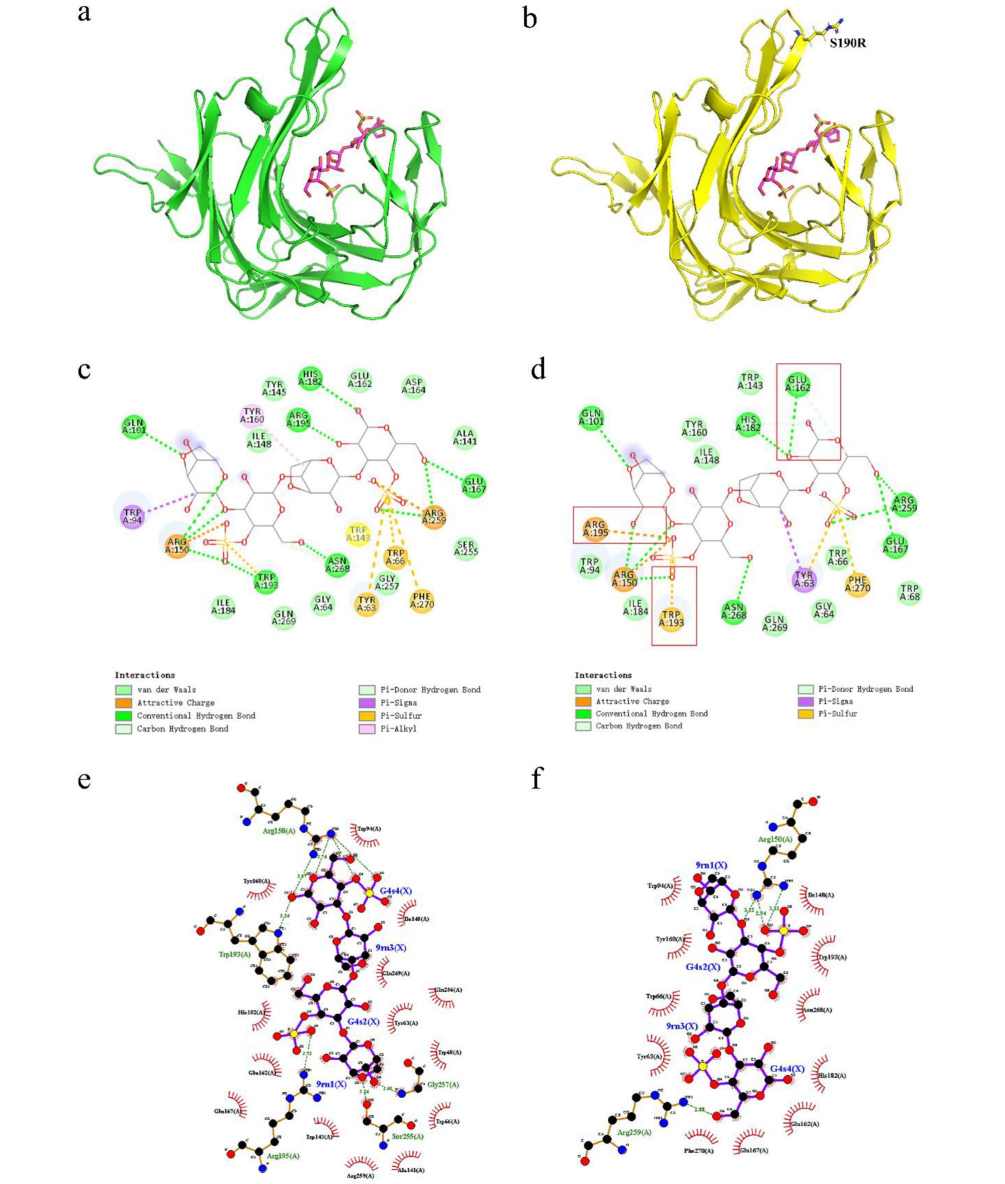
Molecular docking of κ-carrageenase with κ-neocarratetraose. **(a–b)** Molecular docking for WT **(a)** or S190R **(b)** with κ-neocarratetraose. **(c–d) **Two-dimensional force analysis diagrams for WT **(c)** or S190R **(d)** with κ-neocarratetraose. The changed interaction forces were shown in the red boxes in Fig. 5d. **(e–f)** Analysis of hydrogen bonding and hydrophobic interactions between WT **(e)** or S190R** (f) **with κ-neocarratetraose

Protein-ligand interactions can be predicted by the molecular docking (Morris and Della Corte 2021; Kamel et al. 2022). The interactions between WT or S190R and κ-neocarratetraose were shown as 3D schematics (Fig. 5a and b) and 2D schematics (Fig. 5c and d). As shown in Fig. 5c, κ-neocarratetraose interacted with 8 residues in WT (Q101, R150, E167, H182, W193, R195, R259, and N268) through 11 hydrogen bonds. S190R showed an increase of one hydrogen bond with the substrate at residue E162, while reducing two hydrogen bonds at residues W193 and R195 (Fig. 5d). Compared to that of WT (Fig. 5e), the hydrophobic interactions in the S190R-substrate complex were reduced (Fig. 5f). The changes in hydrogen bonding as well as other interactions might result in the changes in thermostability of S190R.

### Molecular dynamics and conformational change analysis of S190R

**Fig. 6:**
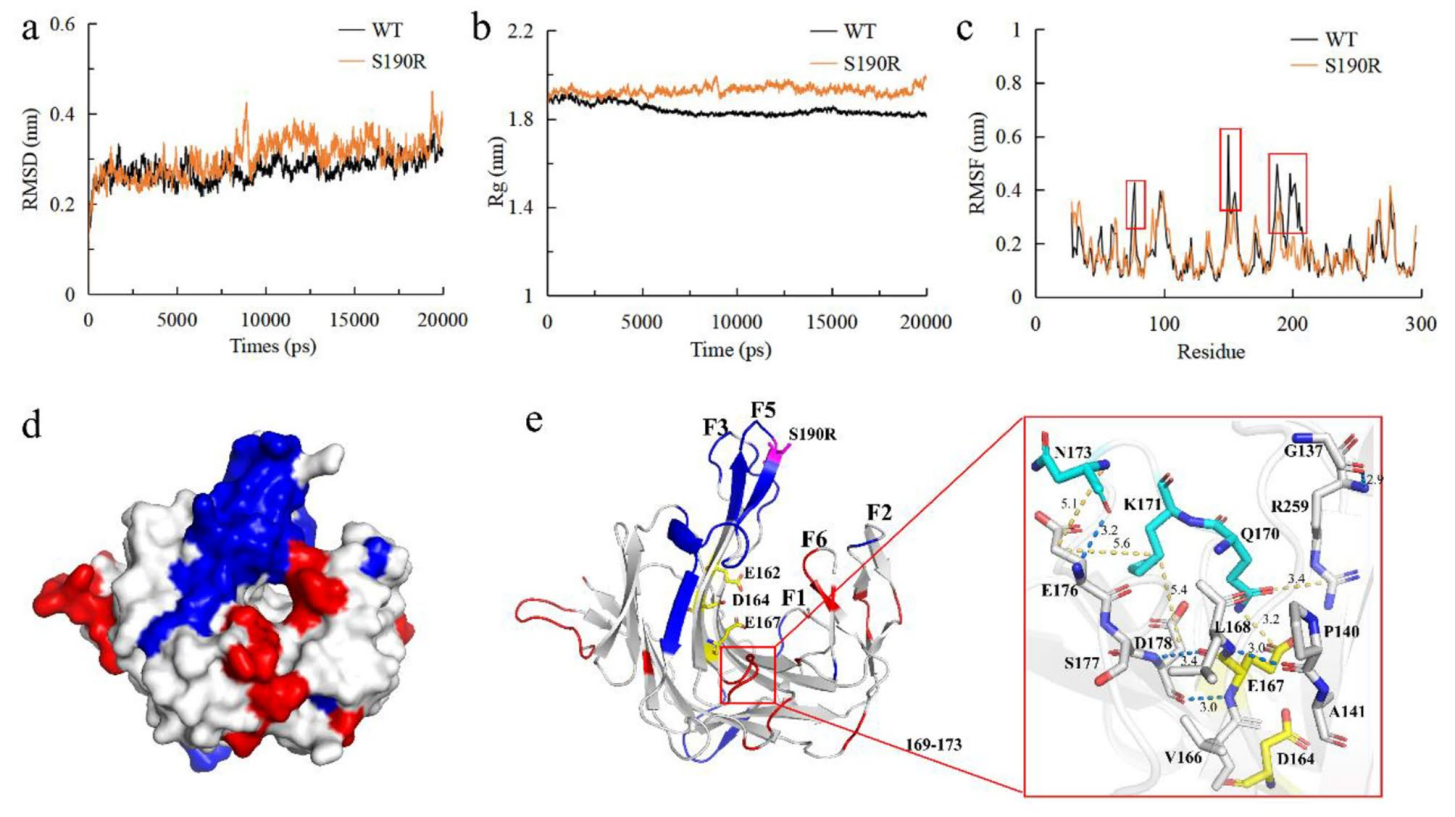
Molecular dynamics and conformational stability analysis of κ-carrageenase. **(a–c)** RMSD **(a)**, Rg **(b)**, and RMSF **(c)** of S190R and WT at 323 K. **(d and e)** The flexibility changes of S190R relative to WT at 323 K (the changes exceeded 0.05 nm). Red was the area where flexibility increases, and blue was the area where flexibility decreases. The catalytic triad was represented by yellow sticks, while the residues with flexibility increased (Q170, K171 and N173) were represented by blue sticks in Fig. 6e

The MD was performed for S190R and WT at 323 K to predict the impact of residue substitution on enzyme conformational stability. As shown in Fig. 6a, the average RMSD values for S190R and WT were 0.30 nm and 0.28 nm at 323 K, respectively. The R_g_ is defined as the average distance from the collection of atoms to their common center of mass (Biswas et al. 2020). At 323 K, the overall average R_g_ value of WT (1.84 nm) was smaller than that of S190R (1.92 nm) (Fig. 6b). Some areas with significant decrease in RMSF values for S190R were displayed in Fig. 6c (represented by red boxes). Among them, the RMSF values near the mutation site 190 were significantly lower than that of WT. With the reference to the RMSF of WT at 323 K, the regions where the structure flexibility of S190R increased and decreased were represented in red and blue, respectively (Fig. 6d and e). S190R had increased the RMSF values in some loops such as P29–T37, T169–N173, and N268–F270 (Fig. 6e). The residues of T169–N173 were near the catalytic center. By Ring-3.0 server analysis, the van der Waals force was found between Q170 and the catalytic residue E167 (Fig. 6e). The increased flexibility of Q170 might affect the thermostability and catalytic activity of mutant S190R. The important residue R259 stabilizing the substrate was also found to be more flexible. This might be responsible for the increased thermostability of S190R. In addition, the flexibility of some loops (D149–D157, N188–G189, and P196–A199), β-sheets (T74–S76, I184–K187, S190–M194, H204–H208), and α-helix (P200–N203) in S190R was significantly reduced (Fig. 6e). Residue R150 located in F3 was predicted to be involved in ion interactions between enzyme and polysaccharide sulfate-ester substituents (Matard-Mann et al. 2017). The F5 (V186–G197) and F6 (A260–P272) fingers were predicted to constitute the closed part of the catalytic tunnel (Matard-Mann et al. 2017). The rigidity of most residues (V186–M194 and P196–G197) in F5 finger was improved significantly after the mutation. The increased rigidity in the F5 finger and the key residue R150 might attributed to the improvement in thermostability of the mutant S190R.

**Fig. 7:**
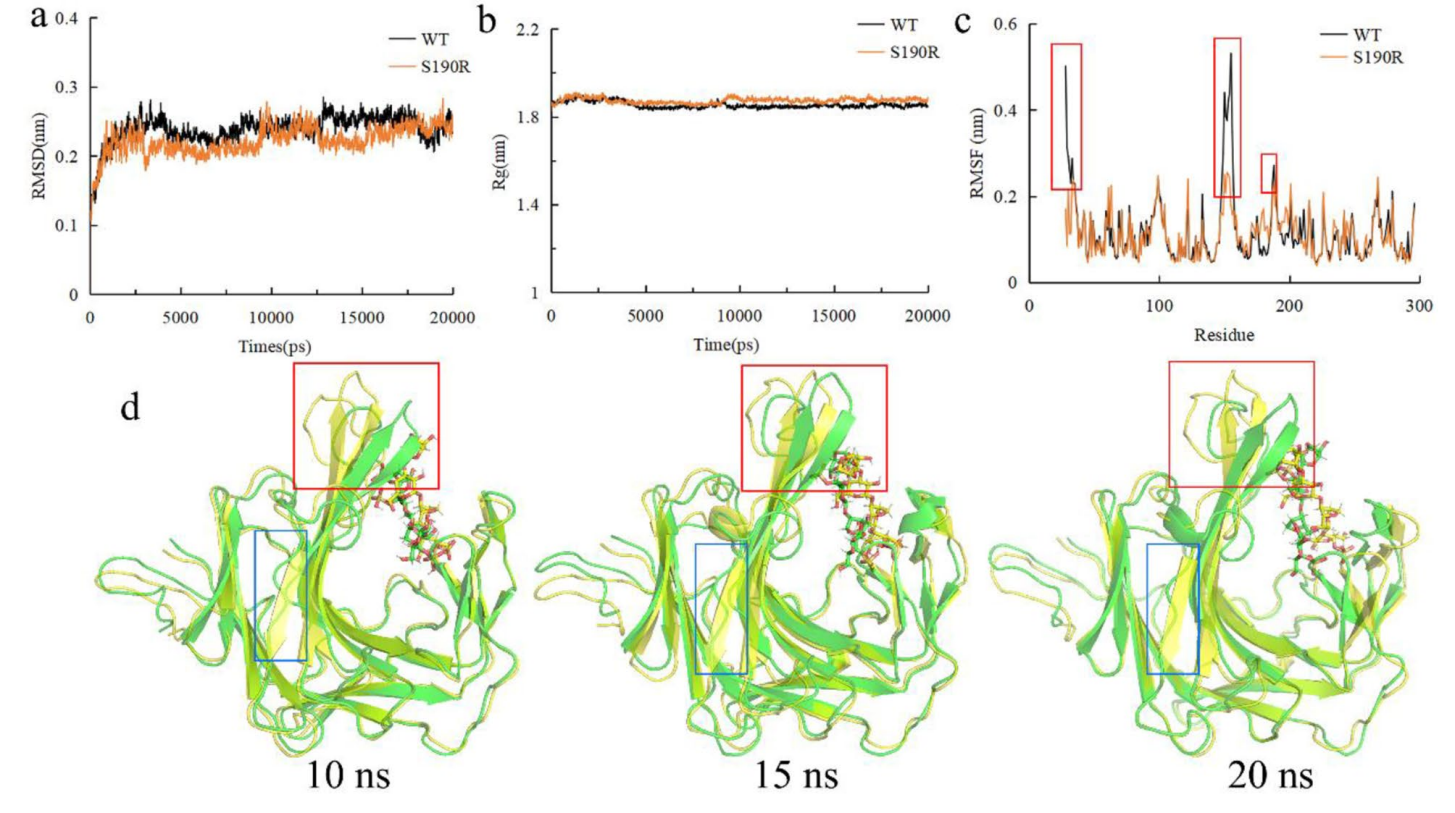
Molecular dynamics and conformational stability analysis of κ-neocarratetraose-enzyme complexes. **(a–c)** RMSD **(a)**, Rg **(b)**, and RMSF **(c)** at 323 K. **(d) **Structural alignment between WT and S190R at three key simulation timepoints of 10 ns, 15 ns, and 20 ns. The green model represented WT, and the yellow one represented the S190R.

MD simulations on the κ-neocarratetraose-enzyme complex at 323 K was performed to predict the conformational stability of WT and S190R when they bound to the substrate. The RMSD and Rg of WT-neocarratetraose and S190R-neocarratetraose were not significantly different (Fig. 7a and b). After combination with the κ-neocarratetraose substrate, the flexibility of S190R was significantly reduced compared to that of WT (Fig. 7c). Specifically, S190R exhibited a significant rigidity increase at residues Q28–K33 (located in the loop region), I148–D157 at F3, and V186–T192 at F5 (represented by red boxes in Fig. 7c). Through the MD simulation trajectory analysis, it was found that the enhancement of F5 rigidity contributed to the stability of the S190R tunnel (represented by red boxes in Fig. 7d), and some structures in the κ-carrageenase underwent significant local unfolding during the simulation process (Fig. 7d). For example, for the original structures of WT and S190R, there was β-sheet at residues H204-H208. However, this sheet was lost at all the three time points (10 ns, 15 ns, and 20 ns) in WT, but it remained folded in S190R (represented by blue boxes in Fig. 7d). This increased local unfolding would weaken the interactions between WT and the substrate, leading to the instability of the entire κ-carrageenase structure (Yu and Dalby 2018).

## Discussion

FoldX is often used in protein stability prediction, and it has shown good performance in screening of heat-resistant mutants (Buß et al. 2018a, b). The MD simulation is a widely used method for predicting protein flexibility, and the RMSF value is used to represent the overall flexibility of the system (Yu and Huang 2014). Making the flexible sites rigid has been shown to be significantly effective in improving the thermostability of proteins (Mamonova et al. 2013). In this study, the mutant κ-carrageenase S190R with improved thermostability was obtained by the combination of FoldX and MD simulation for the alkaline κ-carrageenase KCgCD from *Pseudoalteromonas porphyrae*. The rational design based on ΔΔG calculation and flexibility analysis would be a very effective strategy for improving the κ-carrageenase’s thermostability, facilitating the industrial application of κ-carrageenase S190R for the production of biologically functional κ-carrageenan oligosaccharides. This strategy has also been successfully applied in improving the thermostability of the lipase from *Rhizopus chinensis* (Wang et al. 2020). In addition, the optimal reaction temperature for S190R was 10 °C higher than that of WT. The improvement of the optimum reaction temperature of S190R is crucial to its biocatalysis application under the extreme conditions (Maffucci et al. 2020). The mutation reduced the affinity between enzyme and substrate, but increased the maximum catalytic reaction rate. The improved *V*_max_ promoted the industrial application of S190R.

Due to the different mechanisms of the degradation processes, the κ-carrageenan oligosaccharides prepared by different methods have different chemical structures (Kalitnik et al. 2013). The κ-carrageenase can hydrolyze the β-1,4 linkages in the κ-carrageenan to produce a series of homologous even-numbered oligosaccharides –(An-G4S)_n_–, with 3,6-anhydro-α-D-galactose (An) at the non-reducing end and β-D-galactose-4-sulfate (G4S) at the reducing end (Sun et al. 2014). In this study, the enzymatic degradation products of κ-carrageenan by S190R were disaccharides and tetrasaccharides, and the degradation mode of the enzyme did not alter by the mutation. The κ-carrageenases from different microorganisms may produce different oligosaccharides. For example, the κ-carrageenase from *Cellulophaga lytica* can first hydrolyze κ-carrageenan into octasaccharides and hexasaccharides, and then digest the octasaccharides into disaccharides and hexasaccharides (Yao et al. 2013). The degradation products of *Pedobacter hainanensis* κ-carrageenase is mainly tetrasaccharide (Sun et al. 2014), while the counterparts of κ-carrageenase PLJ30 from *Pseudoalteromonas carrageenovora* ASY5 are mainly disaccharides and tetrasaccharides (Xiao et al. 2018). The biological activities of carrageenan oligosaccharides depend on the number of subunits (Duan et al. 2016).

The xanthine oxidase (XOD) is a molybdenum containing enzyme which is critical in purine catabolism (Mohos et al. 2019). Overproduction of uric acid by XOD from purine compounds or underexcretion of uric acid can lead to hyperuricemia as gout (Jang et al. 2014). Gout is a serious disease that can lead to the formation of urate crystals and their deposition in joints and kidneys (Dalbeth et al. 2021; Haidari et al. 2008). Inhibition of XOD activity is helpful for the reduction of vascular oxidative stress and decrease of uric acid level (Kostić et al. 2015). In this study, the κ-carrageenan oligosaccharides showed stronger inhibitory effects against XOD activity compared with κ-carrageenan. The increased xanthine oxidase inhibitory activity of the κ-carrageenan hydrolysates suggested that the mutant κ-carrageenase S190R would be of great value in the food and pharmaceutical industries (Zhu et al. 2021b).

The molecular dynamics simulation is a unique and powerful tool for predicting the motion and interatomic interactions as a function of time in a protein or bimolecular system (Duran et al. 2021). RMSD is used to measure the distance between two aligned objects and describe the changes of protein secondary structure during simulation (Zhu et al. 2008). The small difference in overall RMSD of S190R and WT might be attributed to the fact that the mutation was located far from the substrate binding site (Rani et al. 2022). RMSF is a measure of the displacement of amino acid residues around their average position within a defined time period, and it can detect the highly flexible regions in proteins (Biswas et al. 2020). The low RMSF value indicates that the residue has low flexibility during the simulation process, having strong stiffness and stability (Madeddu et al. 2022). In this study, the improved rigidity of F5 finger and R150 residue, the increased flexibility of some loops and R259 residue in S190R could account for the improvement of thermostability of the mutant κ-carrageenase.

## Data Availability

All data generated and analyzed during this study are included in this published article.
